# Extracellular Mitochondria Signals in CNS Disorders

**DOI:** 10.3389/fcell.2021.642853

**Published:** 2021-03-05

**Authors:** Ji-Hyun Park, Kazuhide Hayakawa

**Affiliations:** Neuroprotection Research Laboratory, Departments of Radiology and Neurology, Massachusetts General Hospital and Harvard Medical School, Charlestown, MA, United States

**Keywords:** extracellular mitochondria, central nervous system, mitochondrial transfer, biomarker, neurovascular unit

## Abstract

Mitochondria actively participate in the regulation of cell respiratory mechanisms, metabolic processes, and energy homeostasis in the central nervous system (CNS). Because of the requirement of high energy, neuronal functionality and viability are largely dependent on mitochondrial functionality. In the context of CNS disorders, disruptions of metabolic homeostasis caused by mitochondrial dysfunction lead to neuronal cell death and neuroinflammation. Therefore, restoring mitochondrial function becomes a primary therapeutic target. Recently, accumulating evidence suggests that active mitochondria are secreted into the extracellular fluid and potentially act as non-cell-autonomous signals in CNS pathophysiology. In this mini-review, we overview findings that implicate the presence of cell-free extracellular mitochondria and the critical role of intercellular mitochondrial transfer in various rodent models of CNS disorders. We also discuss isolated mitochondrial allograft as a novel therapeutic intervention for CNS disorders.

## Introduction

Mitochondria are the powerhouse of cells and essential for maintaining cellular function in mammals (Devine and Kittler, 2018). The role of mitochondria is especially important in a high-metabolic-rate organ like the brain. Mitochondria produce adenosine triphosphate (ATP) (Jonckheere et al., 2012; Murphy et al., 2019), play a central role in fatty acid biosynthesis (Kastaniotis et al., 2017) and cellular calcium homeostasis (David and Barrett, 2003; Pivovarova and Andrews, 2010), and also regulate intracellular mechanisms that modulate viability, immune cell activation, and mitophagy (Tait and Green, 2012). Accumulating mitochondrial reactive oxygen species (ROS) and inflammasome along with imbalanced mitochondrial membrane permeability may cause progression of cell death and neuroinflammation (Green et al., 2011; Angelova and Abramov, 2018). Therefore, restoring mitochondrial perturbation within cells is a major therapeutic strategy in many CNS disorders including stroke (Rutkai et al., 2019), hemorrhagic stroke (Wang et al., 2018), spinal cord injury (Gollihue et al., 2018a), Alzheimer’s disease (AD) (Wang et al., 2020), and Parkinson’s disease (PD) (Requejo-Aguilar and Bolanos, 2016).

Recent studies in rodents and humans demonstrate that mitochondria may be secreted into the extracellular milieu and transported or exchanged between cells in the CNS (Spees et al., 2006; Islam et al., 2012; Hayakawa et al., 2016; Sinha et al., 2016; Yao et al., 2018; Gao J. et al., 2019; Gao L. et al., 2019; Liu et al., 2019; Marlein et al., 2019; Miliotis et al., 2019; Al Amir Dache et al., 2020; Eun Jung et al., 2020; Park et al., 2020b). Under pathophysiological conditions in the CNS, extracellular mitochondria and their components might contribute to signals between cells that could evoke detrimental inflammation or promote beneficial neuroprotection. In this regard, intact and active extracellular mitochondria may provide a novel therapeutic intervention to restore the bioenergetic needs of damaged or diseased recipient cells. In this mini-review, we collected studies of endogenous mitochondrial transfer as interdependent signals between cells, discuss extracellular mitochondria and its components as a novel class of mediators and biomarkers of injury or disease, and ultimately explore the clinical relevance of mitochondrial transplantation as a therapeutic approach for CNS disorders.

## Mechanisms of Experimental Mitochondrial Transfer

Mitochondria support energy homeostasis in cells. But emerging evidence implicates that mitochondria are surprisingly transferred or exchanged between cells. The biological process was initially discovered in an *in vitro* cell culture system wherein mitochondrial DNA (mtDNA) from human mesenchymal progenitor cells (hMSCs) was delivered to A549 ρo cells (Spees et al., 2006). Similar observations have been subsequently reported by other research groups that showed evidence of mitochondrial transfer between progenitor cells and damaged cells *via* the formation of tunneling nanotubes (TNTs) (Onfelt et al., 2006; Kesner et al., 2016). The TNT-dependent mitochondrial transfer has been suggested in human-originated endothelial progenitors (Koyanagi et al., 2005) and MSCs but not in fibroblast (Yang et al., 2016) and rescued UV stress damage in PC12 cells, cigarette smoke-induced lung damage, and acute respiratory distress syndrome in rodents (Li et al., 2014; Wang and Gerdes, 2015; Jackson et al., 2016).

Extracellular vesicles (EVs) or exosomes can also deliver intracellular mitochondria or the components to other cells. It has been shown that glioblastoma cells and astrocytes produced exosome-enclosed mtDNA, supporting the idea that mtDNA may be delivered to cells through exosomal transfer (Guescini et al., 2010). In the retina, the vesicle-mediated transfer of mitochondria allows neurons to eliminate non-functional mitochondria within astrocytes *via* transmitophagy while these molecular mechanisms are still understudied (Davis et al., 2014). On contrary, astrocyte-to-neuron mitochondrial transfer has been reported as neuroprotection mechanism that reactive astrocytes after stroke produced extracellular mitochondria through CD38-cADPR signaling and these mitochondria were able to enter damaged neurons through an integrin-src/syk pathway to rescue damaged neurons (Babenko et al., 2015; Hayakawa et al., 2016).

It is important to note that the beneficiality of mitochondrial transfer may depend on context. For instance, activated astrocytes after focal cerebral ischemia may express regenerative genes (A2), whereas neurodegenerative diseases activate proinflammatory phenotype (A1) (Zamanian et al., 2012; Khakh et al., 2017; Liddelow et al., 2017; Trias et al., 2018; Yun et al., 2018). Inflammatory microenvironment where A1 astrocytes are enriched also increases proinflammatory microglia, and the crosstalk between inflammatory glia may exacerbate neurodegeneration through intercellular crosstalk *via* disrupted and fragmented mitochondria (Joshi et al., 2019). Collectively, extracellular mitochondria produced by glia can also be deleterious by expanding neuroinflammation and rigorous assessment of the functional property may provide a glimpse into the severity of neurodegeneration.

Although the molecular mechanisms underlying mitochondrial release and internalization remain to be fully elucidated, several pathways are being actively investigated. Recent proof-of-concept studies demonstrate that CD38 signaling may regulate mitochondrial transfer (Hayakawa et al., 2016; Huang et al., 2019; Lippert and Borlongan, 2019; Sun et al., 2019). Stimulating a CD38 downstream with cADPR or amplifying CD38 expression increased functional extracellular mitochondria secreted from astrocytes (Hayakawa et al., 2016). When astrocytes had a mutation R239C in GFAP gene, astrocyte-mediated mitochondrial transfer to neurons was disrupted accompanied by decreasing CD38 expression (Gao L. et al., 2019). What remains missing from the collective literature is the mechanism of how mitochondria are able to maintain their functionality. It has been proposed that mitochondrial protein posttranslational modification may support the functionality outside cells and be a key mechanism to support mitochondrial energy production, mitochondrial membrane potential, and motility in recipient cells postmitochondrial transfer (Yuzwa et al., 2012; Wang et al., 2016; Yang and Qian, 2017; Park et al., 2020a). A proof-of-concept study has been reported. When astrocytic CD38 was stimulated by NAD+, O-GlcNAc posttranslational modification in mitochondrial proteins was amplified, and these O-GlcNAcylated mitochondria maintained mtDNA content and membrane potential in extracellular space, thus improving neuroprotective effects after mitochondrial transfer (Park et al., 2020b).

**FIGURE 1:**
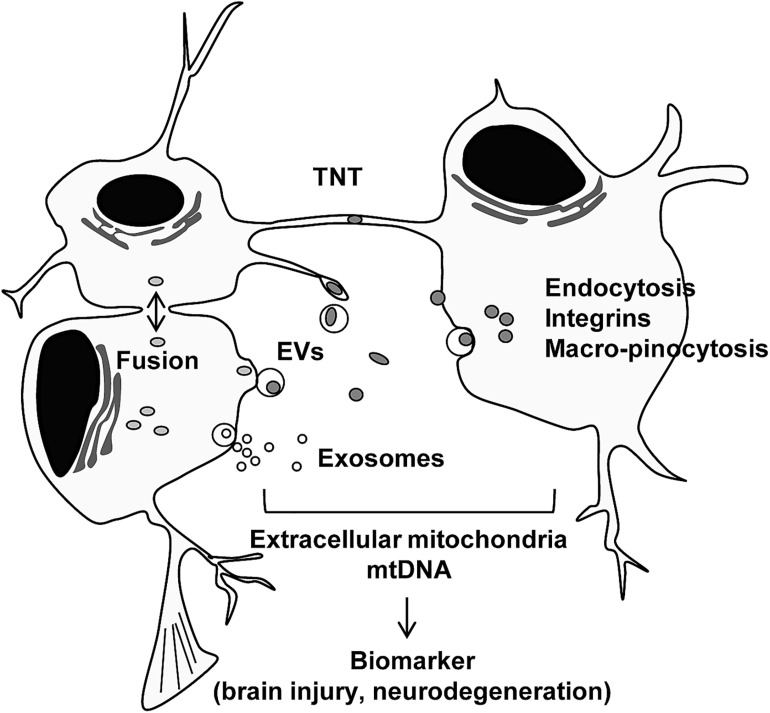
Intercellular mitochondrial transfer. Intercellular mitochondrial transfer can be regulated by tunneling nanotubles (TNT), extracellular vesicles (EVs), exosomes, and cell fusion. Extracellular mitochondria may be incorporated into cells through canonical endocytosis pathway, integrin-mediated pathway, and macro-pinocytosis. Extracellular mitochondria and mtDNA may provide a glimpse into the status of tissue metabolism, disease severity and recovery in the CNS.

Other intracellular mechanisms have been studied. The interaction between mitochondria and the ER may facilitate mitochondrial transfer within the osteocyte dendritic network (Gao J. et al., 2019). Moreover, gap junction protein connexin 43 or mitochondrial transport coordinator Miro 1 may be involved in mitochondrial transfer mechanisms regulated by mesenchymal stem cells (Islam et al., 2012; Ahmad et al., 2014). Cellular stress or stimulus can also trigger mitochondrial secretion and transfer. It has been reported that stressed cells that had lost cytochrome *c* triggered mitochondrial transfer to prevent apoptosis in PC12 cells subjected to UV exposure (Wang and Gerdes, 2015). ROS produced during oxidative stress have been suggested to trigger the secretion of mitochondria (Islam et al., 2012; Torralba et al., 2016; Zhang et al., 2016). TNT was produced in induced pluripotent stem cells (iPSCs) by tumor necrosis factor alpha (TNF-α) and the microstructure interacted with cardiomyocytes through TNFαIP2 expression, which may promote the effective transfer of mitochondria (Zhang et al., 2016). Furthermore, when extracellular mitochondria were internalized into the cells, activation of several pathways including endocytosis (Wei et al., 2018), integrin-dependent pathways (Hayakawa et al., 2016), macro-pinocytosis (Kitani et al., 2014), TNT, or cell fusion (Spees et al., 2006; Torralba et al., 2016) have been observed. Future studies are warranted to identify the specific internalization pathway when extracellular mitochondria are either healthy or disrupted under pathophysiological conditions (Figure 1).

## Extracellular Mitochondria as a Biomarker for Mitochondrial Integrity in the CNS

Under normal conditions, healthy mitochondria maintain their functionality through continuous fission and fusion cycles. During injury or disease, the disruption of the process to integrate damaged mitochondria into the mitochondrial homeostatic system subsequently activates the process of mitochondrial elimination *via* mitophagy, mitochondria-specific autophagy that is a subtype of autophagy regulated by autophagosomes and lysosomes (Ashrafi and Schwarz, 2013), and recovered amino acids and fatty acids following the degradation process can be recycled to generate ATP (Twig et al., 2008; Nakatogawa et al., 2009), supporting that mitophagy is involved in maintaining mitochondrial quality and metabolic status.

The similar mitochondria elimination mechanism may be present in neuron–glial network in retina (Davis et al., 2014). In this study, electron microscopy and confocal microscopy determined that retinal ganglion cells transferred mitochondria-contained vesicles to retinal astrocytes to eliminate damaged mitochondria through so called transmitophagy. In the context of PD, the mechanism of transmitophagy may be crucial to maintain mitochondrial function in dopaminergic neurons and prevent neuroinflammation mediated by disrupted mitochondrial secretion (Morales et al., 2020). In this study, Morales and colleagues observed that damaged dopaminergic neurons *via* 6-OHDA infusion generated spheroid-like structure containing mitochondria and transferred them to adjacent astrocytes for digesting damaged mitochondria through STX17/Lamp1/Lamp2/LC3+ autophagolysosomes (Morales et al., 2020). Collectively, the transmitophagy may be a mechanism of mitochondrial quality control through intercellular mitochondrial transfer in the CNS.

Secreted mitochondria and its components in blood and cerebrospinal fluid (CSF) can be considered promising biomarkers for injury or disease. The basic hypothesis is that extracellular mitochondrial functionality reflects intracellular metabolism and can be indirectly assessed the underlying tissue metabolic integrity (Hayakawa et al., 2018; Miliotis et al., 2019). In CSF analysis in subarachnoid hemorrhage (SAH), membrane potentials of extracellular mitochondria were decreased in SAH patients, and higher JC1 ratios were associated with better clinical recovery at 3 months after SAH (Chou et al., 2017; Youn et al., 2020). Cell-free mtDNA becomes a damage-associated molecular pattern (DAMP) acting as a “danger signal” (Galluzzi et al., 2012; Thurairajah et al., 2018). Therefore, the amount of mtDNA in CSF may also implicate inflammation status. In fact, higher mtDNA contents in CSF were correlated to the progression of AD (Cervera-Carles et al., 2017) or anti-NMDA receptor encephalitis (Peng et al., 2019).

Altogether, extracellular mitochondria and mtDNA may be a biomarker for mitochondrial integrity, disease progression, or recovery in CNS pathology including neuroinflammation, hemorrhagic stroke, and neurodegenerative disease (Figure 1). Additional studies are needed to address whether the concept of extracellular mitochondria as a biomarker is applicable in other CNS disorders such as spinal cord injury and PD.

## Mitochondrial Transplantation as CNS Therapies

**FIGURE 2:**
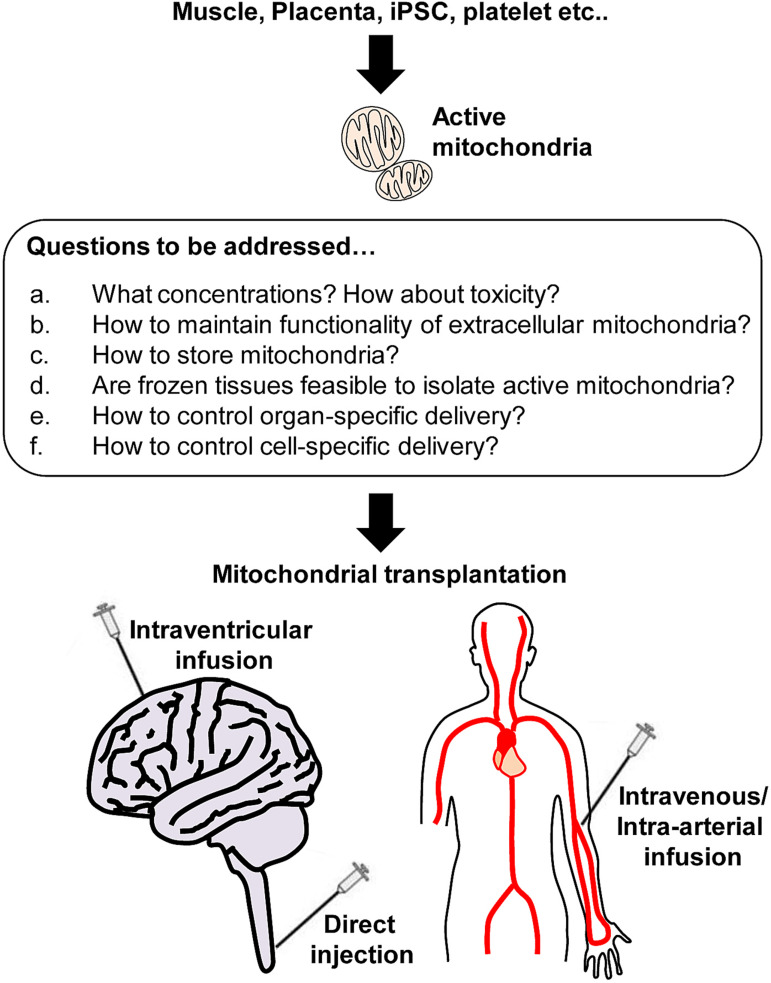
Active mitochondria isolation from various sources and beyond. Respiratory active mitochondria are able to isolate from various sources including skeletal muscle, placenta, iPSC, and platelets. However, many questions remain to be answered regarding mitochondrial transplantation. a: It is critical to identify the optimal concentration of mitochondria that do not induce toxicity. b: Protecting extracellular mitochondria against pathological environments (high calcium, ionic imbalance, high ROS, glycation etc.) would be challenging, but it is required to avoid disrupting mitochondria while maximize the benefit induced by mitochondrial transplantation. c-d: Storing mitochondria is the most challenging issue. Instead, it might be worth seeking if frozen tissues can be sources to isolate a larger amount of active mitochondria. e-f: Finally, it is critical to investigate how to deliver mitochondria to target tissues or cells to maximize the therapeutic efficacy.

In the CNS, neuron-glial-vascular interaction is essential for a homeostatic network, the so-called neurovascular unit in the CNS (Iadecola, 2004; Lo et al., 2004; Hawkins and Davis, 2005; Zacchigna et al., 2008; Zlokovic, 2008; del Zoppo, 2009) and functional impairment of mitochondria within the unit can be one of the major reasons to cause CNS disorders. McCully and colleagues have studied mitochondrial transplantation to the heart as a promising therapy without inducing detrimental immune response (McCully et al., 2016; Shin et al., 2019). These studies have supported scientists to investigate therapeutic efficacy utilizing exogenous mitochondria for CNS injury or neurodegeneration. Accumulating evidence has shown the beneficial actions of mitochondrial transplantation and the limitation in various animal models of CNS disorders (Figure 2).

### Spinal Cord Injury

The therapeutic potential of extracellular mitochondria has been reported in a mouse model of spinal cord injury (SCI) (Gollihue et al.,2018a,b). In this study, GFP-labeled mitochondria (50–150 μg) were isolated from PC12 cells and then transplanted into the injury site after SCI onset. Following direct microinjection, macrophages, endothelial cells, and astrocytes incorporated injected mitochondria accompanied by improving acute mitochondrial bioenergetics and mitochondrial respiration (Gollihue et al., 2018a). Intriguingly, injected mitochondria were not found in neurons and did not support functional neuroprotection, implicating that mechanisms of mitochondrial internalization may be different among cell types. Ultimately, the study provides a very important aspect that mitochondrial incorporation efficiency in neurons may need to be improved to accelerate functional recovery after SCI.

Kuo and colleagues attempted to prevent axonal degeneration by mitochondrial transplantation in a rat model of sciatic nerve crush injury. In this study, BHK cell-derived mitochondria were utilized for therapeutic intervention. Dosages of mitochondria (0, 65, 130, 195, and 269 μg) were initially assessed in the cultured sciatic nerve. After the determination, extracellular mitochondria (195 μg) were injected directly into the injured nerve. Notably, mitochondria transplantation increased neuronal regeneration and decreased oxidative stress along with improving functional behaviors and physiological neuronal and muscle activities. Moreover, mitochondrial transplantation increased the expression of neurotrophic factors such as BDNF and CNTF in injured nerves along with restoring muscular integrity and increasing muscle progenitors and total muscle mass (Kuo et al., 2017).

### Parkinson’s Disease

Many genes involved in familial PD have been identified and known to directly link to mitochondrial dysfunction (Lill, 2016). The impairment of the respiratory chain, redox homeostasis, mitophagy, and mitochondrial biogenesis in dopaminergic neurons may aggravate PD pathogenesis (Moon and Paek, 2015). Given the fact that mitochondria-targeting antioxidants such as coenzyme Q10 and creatine monohydrate did not effectively ameliorate PD pathology (Beal et al., 2014; Kieburtz et al., 2015), healthy mitochondrial transplants to the damaged brain area can be an alternative approach to recover mitochondria-mediated redox homeostasis within neurons if these mitochondria can successfully incorporate into disrupted cells.

A study has been conducted using a mouse model of MPTP-induced PD. Fluorescent-labeled mitochondria were delivered systemically *via* tail vein injections in this model. Intriguingly, infused mitochondria (IV route, 0.5 mg/kg body weight) were ubiquitously found in CNS tissues and peripheral organs. Most importantly, mitochondrial infusion significantly improved motor functions accompanied by recovering mitochondrial complex I activity and ATP production, thus inhibiting cell death pathways in the striatum in comparison with the vehicle group (Shi et al., 2017), suggesting that intravenous infusion of mitochondria may be feasible to target not only peripheral organs but also CNS tissues.

Another study has utilized a mitochondrial modification technique with Pep-1 to improve mitochondrial delivery into CNS tissues. Interestingly, mice treated with Pep-1-conjugated mitochondria into the substantia nigra pars compacta (1.05 μg/5 μl) showed better behavioral outcomes in locomotor activity, moving distance, and moving speed compared to ones treated with unmodified control mitochondria (Chang et al., 2016). Concomitantly, mitochondrial incorporation in TH+ dopaminergic neurons was improved, suggesting that mitochondrial modification can amplify the therapeutic potential of exogenous mitochondria and the beneficiality to enhance neuronal incorporation in the CNS.

### Alzheimer’s Disease

Alzheimer’s disease is a neurodegenerative disease along with severe memory loss with impairment of episodic memory in the initial phases (Swerdlow et al., 2014). Accumulating evidence indicates that mitochondrial impairment may contribute to the pathology of AD. Indeed, brain mitochondria in AD have shown dysfunctional respiration chain complexes, decreased ATP generation, and an excessive amount of free radicals that may lead to neurodegeneration (Parker and Parks, 1995; Pohland et al., 2018). In addition to therapies attacking amyloid-beta, targeting mitochondrial dysfunction may indirectly ameliorate AD pathology. Nitzan and colleagues have attempted mitochondrial transplantation to restore mitochondrial function in a mouse AD model produced by Aβ 1–42 intracerebroventricular infusion (Nitzan et al., 2019). Mitochondria isolated from HeLa cells were injected through the tail vein. As a result, IV-injected mitochondria were observed in the liver, but there was no signal in the brain following IV injection. Intriguingly, mitochondrial infusion still improved cognitive functions accompanied by decreasing loss of neurons and suppressing hippocampal glial activation. Moreover, mitochondrial functional parameters in the liver and brain were also restored. Collectively, mitochondrial transplantation through IV route can be a new therapeutic strategy for AD (Nitzan et al., 2019).

Mitochondrial transplantation also improved diabetes-associated cognitive impairment (DACI) which is associated with elevated Aβ deposition (Ma et al., 2020). In this study, platelet-derived mitochondria were used as the donor source. After intracerebroventricular injection of mitochondria in db/db mice, mitochondrial internalization to hippocampal neurons was observed, and subsequently, DACI was alleviated accompanied by restored mitochondrial function as well as decreased accumulation of Aβ. Although further rigorous assessments are required, it may be promising that mitochondrial transplantation may ameliorate AD pathology *via* inhibiting Aβ generation and deposition.

### Ischemic Stroke

Therapeutic interventions including recombinant tissue plasminogen activator or endovascular thrombectomy provide effective ways to achieve reperfusion for acute ischemic stroke (Vosler et al., 2009). While restoring cerebral blood flow is essential for decreasing infarction, reperfusion alone may not be sufficient to fully salvage penumbral tissue in some patients where cells are dying through active cell death pathways (Lo, 2008). Therefore, it is still important to continue the search for neuroprotective approaches that can be added to ischemia reperfusion (Vosler et al., 2009). The brain with ischemic stroke results in a lack of supplementations for glucose and oxygen and mitochondrial disruption to generate ATP, thus causing mitochondrial metabolic impairment and leading to neuronal apoptosis. Collectively, mitochondrial restoration therapy utilizing extracellular mitochondria may also be applicable in ischemic stroke (Watts et al., 2013; Baek et al., 2014; Russo et al., 2018).

Mitochondrial transplantation may indeed provide some beneficial effects to protect neurons in models of ischemic stroke. It has been shown that intact mitochondria isolated from BHK-21 cell lines successfully protected neurons against ischemic insult. In this study, mitochondria were treated directly into the striatum (75 μg) or infused through arteries (750 μg) after transient focal cerebral ischemia. Neurons and glia in the peri-infarct area appeared to incorporate mitochondria treated *via* both routes at 4 weeks after ischemic stroke. Moreover, treatment with isolated mitochondria significantly improved motor function accompanied by decreasing infarction and TUNEL-positive cells in acute stroke (Huang et al., 2016). Treatment with skeletal muscle-derived mitochondria may also provide beneficial neuroprotective effects (Zhang et al., 2019). Freshly isolated mitochondria from skeletal muscle (5 × 10^6^/10 μl) were infused into the lateral ventricle immediately after reperfusion after focal cerebral ischemia. Consistent with prior findings, the peri-infarct neurons incorporated the treated mitochondria. Subsequently, the allograft remarkably attenuated infarct formation accompanied by restoring neurological impairments, reducing oxidative stress response and gliosis, as well as neuronal death at 28 days poststroke. Collectively, mitochondrial allograft may not only protect neurons against acute injury but also improve long-term outcomes after stroke.

Tissue source for viable mitochondrial isolation is important for practical use of mitochondria in therapy. A proof-of-concept study using placenta-derived mitochondria was performed in a mouse stroke model. From the snap-frozen placentae obtained from E17 pregnant female mice, mitochondria were isolated and evaluated the functionality. Surprisingly, flow cytometry analysis demonstrated that up to 87% of placental mitochondria were viable and maintained JC1 membrane potentials after isolation. Placental mitochondrial fractions contained ATP equivalent to mitochondrial fractions isolated from skeletal muscle and brown fat tissue. Moreover, glutathione reductase, Mn-SOD, and HSP70 were highly preserved in placental mitochondrial fractions. Then, placental-derived mitochondrial fractions (100 μg/100 μl) were infused intravenously immediately after reperfusion with full blinding and randomization. Strikingly, treatment with placental mitochondrial fractions significantly decreased infarction after focal cerebral ischemia in mice. Collectively, cryopreserved placenta can be a feasible source for viable mitochondrial isolation and transplantation with placental mitochondria may amplify beneficial effects of reperfusion in stroke (Nakamura et al., 2020).

## Conclusion

Status of mitochondrial function is a key for recovery after CNS injury or disease (Anne Stetler et al., 2013; Madsen et al., 2017). Accumulating evidences implicate that mitochondria can be surprisingly present outside cells and transported from cell to cell. Moreover, extracellular mitochondria are found not only in rodents but also in clinical samples, and assessments of mitochondrial functionality in extracellular fluids may provide us a biomarker-like glimpse into injury or repair/regeneration. Within the emerging paradigm of targeting mitochondria after CNS injury or disease (She et al., 2017; Vekaria et al., 2017; Simon et al., 2019), viable mitochondria may be feasible to isolate and utilized for CNS therapy. Nonetheless, more rigorous studies are required to validate the reliability, safety, and efficacy of extracellular mitochondria in a wide range of applications from diagnosis to therapeutic intervention in various CNS disorders.

## Author Contributions

J-HP and KH collected literature and prepared the manuscript. All authors contributed to the article and approved the submitted version.

## Conflict of Interest

The authors declare that the research was conducted in the absence of any commercial or financial relationships that could be construed as a potential conflict of interest.
